# DNA Repair Gene Polymorphism and the Risk of Mitral Chordae Tendineae Rupture

**DOI:** 10.1155/2015/825020

**Published:** 2015-10-28

**Authors:** Aysel Kalayci Yigin, Mehmet Bulent Vatan, Ramazan Akdemir, Muhammed Necati Murat Aksoy, Mehmet Akif Cakar, Harun Kilic, Unal Erkorkmaz, Keziban Karacan, Suleyman Kaleli

**Affiliations:** ^1^Department of Medical Biology and Genetics, Faculty of Medicine, Sakarya University, 54290 Sakarya, Turkey; ^2^Department of Cardiology, Faculty of Medicine, Sakarya University, 54290 Sakarya, Turkey; ^3^Department of Biostatistics, Faculty of Medicine, Sakarya University, 54290 Sakarya, Turkey; ^4^Department of Anatomy, Faculty of Medicine, Sakarya University, 54290 Sakarya, Turkey

## Abstract

Polymorphisms in Lys939Gln XPC gene may diminish DNA repair capacity, eventually increasing the risk of carcinogenesis. The aim of the present study was to evaluate the significance of polymorphism Lys939Gln in XPC gene in patients with mitral chordae tendinea rupture (MCTR). Twenty-one patients with MCTR and thirty-seven age and sex matched controls were enrolled in the study. Genotyping of XPC gene Lys939Gln polymorphism was carried out using polymerase chain reaction- (PCR-) restriction fragment length polymorphism (RFLP). The frequencies of the heterozygote genotype (Lys/Gln-AC) and homozygote genotype (Gln/Gln-CC) were significantly different in MCTR as compared to control group, respectively (52.4% versus 43.2%, *p* = 0.049; 38.15% versus 16.2%, *p* = 0.018). Homozygote variant (Gln/Gln) genotype was significantly associated with increased risk of MCTR (OR = 2.059; 95% CI: 1.097–3.863; *p* = 0.018). Heterozygote variant (Lys/Gln) genotype was also highly significantly associated with increased risk of MCTR (OR = 1.489; 95% CI: 1.041–2.129; *p* = 0.049). The variant allele C was found to be significantly associated with MCTR (OR = 1.481; 95% CI: 1.101–1.992; *p* = 0.011). This study has demonstrated the association of XPC gene Lys939Gln polymorphism with MCTR, which is significantly associated with increased risk of MCTR.

## 1. Introduction

Although definite cause of chordae tendineae rupture remains confusing, primary rupture is thought to be more frequent cause than the other underlying situations such as mitral valve prolapse, subacute bacterial endocarditis, local myxomatous degeneration, rheumatic heart disease (mostly during the chronic stage), systemic or generalized connective tissue abnormality (such as in Marfan syndrome, osteogenesis imperfecta, and Ehler-Danlos syndrome), blunt chest trauma, hypertrophic cardiomyopathy, other (nonmitral) heart and valvular abnormalities (such as aortic stenosis and aortic regurgitation), ischemic heart disease in general, and myocardial infarction in particular [1].

Primary or idiopathic mitral chordae tendineae rupture (MCTR) is a progressive condition that may eventually require mitral valve surgery, as the patients treated medically suffer a high (6.3%) mortality rate. The vast majority of patients with idiopathic MCTR experience the separation of multiple chords, leading to severe mitral regurgitation and requiring immediate surgical intervention [2, 3]. Understanding the underlying mechanisms involved in the pathogenesis of idiopathic MCTR is vital in the development of novel therapeutic strategies [3, 4]. Despite its clinical importance, the precise pathophysiologic mechanisms of MCTR have not been established. However, it is evident that the altered expression of genes encoding signal cascade which instigate apoptosis, cell differentiation, cardiac remodeling, angiogenesis, and fibrosis may have an important role in the pathogenesis of MCTR [5, 6]. Recently, Kimura et al. revealed that downregulation of tissue inhibitor of metalloproteinase (TIMP) 2 and tenomodulin and upregulation of vascular endothelial growth factor- (VEG) A and matrix metalloproteinases (MMP) likely play a major role in the molecular mechanism underlying the pathophysiology of MCTR [5]. In addition, Lin et al. also showed that MMP 1 gene expression and MMP 1-1607-16/26 gene polymorphism were independently associated with MCTR [6]. These preliminary studies have demonstrated that genetic factors have an influence on individual susceptibility to MCTR.

The Xeroderma Pigmentosum Complementation Group C (XPC) made up one of the eight core genes (i.e., ERCC1, XPA, XPB, XPC, XPD, XPE, XPF, and XPG) and serves as the primary initiating factor in the genome nucleotide excision repair pathway (NER) [7–9]. The XPC gene encodes a 940-amino acid protein, spanning 33 kb on chromosome 3p25, and contains 16 exons and 15 introns [10]. The XPC gene plays a crucial role in maintaining a balance between cell proliferation and apoptosis [11–13]. The XPC gene polymorphism enhances DNA damage-induced apoptosis through the activation of p53 or p53-independent apoptotic pathway [14–16]. Increasing evidence has provided support for the existence of apoptotic pathways and their regulators in the heart [17]. The relationship between the pathways of apoptosis and various valvular disorders, such as degenerative aortic valve disease and myxomatous mitral valve disease, has been demonstrated in previous studies [18, 19]. However, the role of pathways of apoptosis in MCTR remains unknown. This study has been conducted to investigate the role or association of XPC polymorphisms in the pathogenesis of idiopathic MCTR patients.

## 2. Methods

### 2.1. Study Subjects

Twenty-one patients with idiopathic MCTR and thirty-seven subjects without valvular heart disease were recruited from files of the Department of Cardiology of Sakarya University. All subjects in the MCTR group underwent transesophageal echocardiography to confirm the diagnosis. The Ethical Committee of Sakarya University School of Medicine provided ethical approval for this study. The study conformed to the ethical principles of the Declaration of Helsinki. Informed written consent was obtained from each subject included in the present study. Patients with infective endocarditis, rheumatic heart disease, mitral valve prolapse, myxomatous degeneration, connective tissue disease, blunt chest trauma, hypertrophic cardiomyopathy, and acute coronary syndromes including acute myocardial infarction, chronic renal failure, chronic congestive heart failure, known or suspected infiltrative cardiac diseases, constrictive and/or restrictive cardiomyopathy, and chronic-active inflammatory disease, current smokers, and those diagnosed with any cancer were excluded from the study. Clinical and demographical data, such as age, gender, height, and weight, were recorded on patient charts. Measures of cardiovascular risk, including hypertension, diabetes mellitus, and medication use, were also evaluated.

### 2.2. Echocardiographic Examination

In all subjects, transthoracic two-dimensional and Doppler echocardiographic examinations performed by a Vivid 5 echocardiography system (General Electric Vingmed USG, Israel), using 2.5 MHz transducers were carried out according to standards of the American Society of Echocardiography. Transesophageal echocardiography (TEE) was performed in all patients with MCTR. Left atrial (LA) diameter was measured from the parasternal left heart long-axis view. Pulmonary artery trunk and pulmonary flow were measured from the aortic short-axis view. Mitral inflow and aortic valve flow were also measured. Pulmonary artery systolic pressure was calculated according to velocity of tricuspid regurgitation using the Bernoulli equation. The left ventricular end diastolic diameter (LVEDd) and ejection fractions (EF) were calculated by the M-mode method. The quantification of mitral regurgitation (MR) is made according to the echocardiographic methods including vena contracta width, the flow convergence method, and color Doppler characteristics of regurgitant jet penetration. The mitral valve and its chordae tendineae were observed in the left ventricular midesophageal and MV transgastric views, using rotation of the TEE probe to achieve the clearest view. MCTR diagnosis was made on the basis of flail mitral valve associated with chordal elongation and chordal swinging, with or without chordal remnant root.

### 2.3. DNA Isolation and Genotyping

**Figure 1:**
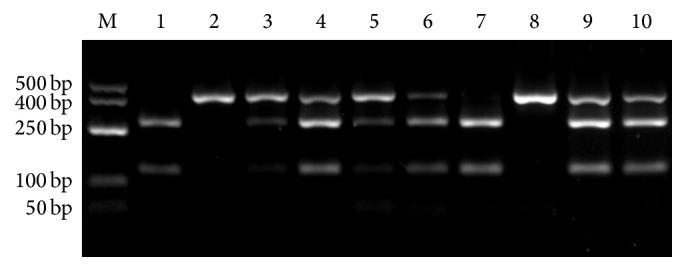
Genotypes were generated from 402 bp PCR product acquired from 9 individuals using specific primers after digestion with PvuII restriction enzyme. The lanes were as follows: M: DNA ladder; lanes 1 and 7: CC genotype, homozygote variant; lanes 2 and 8: AA genotype, homozygote wild type; lanes 3, 4, 5, 6, 9, and 10: AC genotype; heterozygote.

Genomic DNA was extracted from whole blood samples using a special isolation kit (Roche Diagnostics, DNA Isolation Kit for Mammalian Blood Germany). All isolation steps were carefully performed according to the manufacturer's instructions. Genotyping of XPC Lys939Gln polymorphism was carried out using polymerase chain reaction- (PCR-) restriction fragment length polymorphism. PCR amplification was performed in a 30 *μ*L PCR volume containing 100 ng template DNA, 0.750 units Taq polymerase (Fermentas) 1x Mg++ free PCR buffer (Fermentas), 200 mM dNTP (Fermentas), 1.5 mM MgCl++, 10 pMol of each F: 5′-GGCTTCCTGGTATCTGATTACT-3′, R: 5′-CTCAGTTTGCCTTCTCAGCA-3′ (GenBank accession number NC_000003.12). The incubation process of the touchdown-PCR (Sensoquest Thermocycler, Germany) was performed as follows: 4 min at 95°C, followed by 16 cycles for 30 sec at 95°C, 30 sec at 60°C, and 45 sec at 72°C adding 0.5 sec/cycle. Then, 30 cycles of the triple steps were performed for 30 sec at 94°C, 30 sec at 52°C, and 2 min at 72°C; the final elongation step was achieved after 10 min at 72°C. After agarose gel electrophoresis, the 10 *μ*L PCR-amplified DNA was digested with PvuII restriction enzyme for 20 minutes at 37°C and electrophoresed on 2% agarose gel. The homozygote wild-type genotype was identified by a single band at 402 bp level, the heterozygote genotype by 3 bands at 402, 276, and 126 bp levels, and the homozygote variant by 2 bands at 276 and 126 bp levels (Figure 1).

### 2.4. Statistical Methods

Distributions of genotype were suited to the Hardy-Weinberg equilibrium in both control and case groups (*p* = 0.623 and *p* = 0.519, resp.). Pearson's and Fisher's Chi-Square tests were used to compare the genotypes, alleles, and other categorical data between control and case groups. Categorical data were expressed as count and percentages. According to Kolmogorov-Smirnov normality test, two independent-sample *t*-tests were used to compare the continuous variables between two groups. Continuous data were expressed as mean and standard deviation. A multiple logistic regression model was implemented to determine genotypes and other covariates associated with XPC. The odds ratio was calculated for heterozygous and mutant individuals in the assessment of risk status according to the normal group. A *p* value <0.05 was considered significant. Analyses were performed using commercial software (IBM SPSS Statistics, Version 22.0; Armonk, NY: IBM Corp.).

## 3. Results

This study contains 21 MCTR patients with 57 ± 16 mean age and 37 control groups with 52 ± 16 mean age. Of the 21 MCTR patients, 15 (71.4%) were male and 6 (28.6%) were female. Of the 37 control groups, 19 (51.3%) were male and 18 (48.7%) were female. Demographic, echocardiographic, laboratory, and genotype/allele frequencies of the XPC Lys939Gln polymorphisms between MCTR patients and controls are summarized in Tables 1 and 2. The frequencies of the heterozygote genotype (Lys/Gln-AC) and homozygote genotype (Gln/Gln-CC) were significantly different in MCTR as compared to control group, respectively (52.4% versus 43.2%, *p* = 0.049; 38.15 versus 16.2%, *p* = 0.018). The odds ratio for heterozygous and mutant individuals was calculated in the risk status assessment, which is prepared regarding the normal group. Homozygote variant (Gln/Gln) genotype was significantly associated with increased risk of MCTR (OR = 2.059; 95% CI: 1.097–3.863; *p* = 0.018). Heterozygote variant (Lys/Gln) genotype was also highly significantly associated with increased risk of MCTR (OR = 1.489; 95% CI: 1.041–2.129; *p* = 0.049). The variant allele C was found to be significantly associated with MCTR (OR = 1.481; 95% CI: 1.101–1.992; *p* = 0.011) (Table 3).

## 4. Discussion

There are currently no published reports in the literature on the XP polymorphisms profile of MCTR. Thus, this is to our knowledge the first study demonstrating a link between the XPC polymorphisms and MCTR. This study showed that increased heterozygote genotype (AC) and homozygote genotype (CC) frequencies of the XPC Lys939Gln polymorphisms might result in increased genetic susceptibility to MCTR. Furthermore, the variant allele C genotype was also highly associated with increased risk of MCTR.

MCTR is an increasingly important cause of mitral regurgitation and is more common in male adults over 50 [1]. Rupture of a single structure of chordae leads to limited hemodynamic effect and usually requires neither intervention nor treatment. Significant rupture of a multiple structure of chordae tendineae usually results in acute severe mitral regurgitation and hemodynamic instability, requiring prompt surgical intervention [2, 3]. The leading underlying causes in the literature were blunt thoracic trauma, connective tissue diseases, coronary artery disease, infective endocarditis, rheumatic mitral stenosis, and mitral valve prolapse. Undetermined cases are defined as primary (idiopathic) chordae rupture in which there is obviously no underlying cause. In a recent systematic review, the prevalence of idiopathic MCTR was 51.2% [1, 20, 21].

The pathophysiologic mechanism that results in idiopathic MCTR is still unclear. Recently, the investigators have attempted to establish genetic factors, which are thought to play a key role in MCTR occurrence. Evidence for these studies demonstrated that inflammation, increased expression of genes encoding signal cascade which instigate apoptotic balance, and cell differentiation may have an important role in the pathogenesis of MCTR [5, 6].

DNA repair genes protect genomic integrity and they are basic components of normal cell homeostasis necessary to cell growth, differentiation, and apoptosis [21, 22]. It is known that diminished DNA repair capacity, due to various DNA repair gene polymorphisms, is associated with increased risk and susceptibility to human solid tumors [23–25]. XPC is a key member in the nucleotide excision repair (NER) pathway. It is involved in the recognition and initiation of the genome repair of NER pathway [7–9]. Polymorphisms in the XPC gene may alter DNA repair capacity of the NER pathway, which further play a critical role in carcinogenesis. Several polymorphic variants in the XPC gene have been identified. The most common polymorphisms of XPC gene are Ala499Val (C→T), PAT (−/+), and Lys939Gln (A→C) [25–28]. Increasing evidence has accumulated suggesting that XPC gene polymorphism can cause replication blockage, which also enhances unregulated apoptosis induced by DNA damage [11–13]. Recent studies support the claim that apoptosis does occur in the heart [17]. Data obtained from numerous studies have suggested a possible role for apoptosis in heart failure, ischemia/reperfusion injury, idiopathic dilated cardiomyopathy, ischemic cardiomyopathy, arrhythmogenic right ventricular dysplasia, hypertrophic cardiomyopathy, and viral myocarditis [17, 29, 30].

In this study, we hypothesized that unregulated apoptosis induced by reduced DNA repair capacities due to inherited XPC gene polymorphisms may contribute to the development of MCTR. Because of the unique architect and tissue components of chordae tendineae, any changes in DNA damage may eventually lead to MCTR. Recently, experimental and clinical studies also showed a close association between apoptosis and valvular heart disease [18, 19]. A study by Galeone demonstrated that TNF-related apoptosis-inducing ligand is characteristically present within calcific aortic valves and mediates the calcification of aortic valve interstitial cells in culture through mechanism involving apoptosis. Another study by Torigoe et al. has revealed that apoptotic regulatory genes and antiapoptotic mechanism may play a role in the pathogenesis of canine myxomatous mitral valve disease [19]. As yet, the role of apoptosis in the pathogenesis of MCTR has not been evaluated. However, some evidence has supported the possible role of apoptosis on MCTR. This evidence suggests that upregulation of MMP and downregulation of tissue inhibitor of metalloproteinase (TIMP) 2 genes predispose patients to ruptured chordae tendineae [5, 6]. It has become known that MMPs and TIMP 2 may be potential regulators of apoptosis. MMPs cleave cell surface receptors; they release apoptotic ligands like FAS ligand. MMPs affect many behavioral patterns of the cell, such as proliferation, differentiation, migration, and apoptosis. TIMP 2 is an endogenous inhibitor for MMPs. In contrast, TIMP-2 is able to promote cell proliferation in a wide range of cell types and may also have an antiapoptotic function. Based on the above observations, we intended to investigate XPC Lys939Gln polymorphisms and the risk of mitral chordae tendineae rupture. We may report that heterozygote genotype (AC) and homozygote genotype (CC) of XPC gene were highly associated with MCTR. It has also been found that apoptosis induced by DNA damage as a consequence of XPC polymorphism makes people more prone to develop MCTR, as well as cancers. This study provides preliminary results for further studies of the relatively large number of participants. It is hoped that a better understanding of the molecular mechanisms of MCTR may yield novel diagnostic and therapeutic targets.

## 5. Limitations

Our study has some limitations. The major limitation of the present study is its small sample size. Therefore, although the results of our study are interesting, they should be interpreted cautiously, until further studies including a greater number of patients may be conducted.

## 6. Conclusion

Our study has shown that XPC Lys939Gln gene polymorphism is correlated with an increased risk of the MCTR development. Abnormal regulation of apoptosis and diminished DNA repair capacity may be present in the molecular mechanism of MCTR. Further studies with a larger number of patients are warranted to validate these findings.

## Figures and Tables

**Table 1:** Baseline characteristics and laboratory findings of case and control groups.

	Control (*n* = 37)	Case (*n* = 21)	*p*
Genotype			
AA	15 (40.54)	2 (9.52)	**0.026**
AC	16 (43.24)	11 (52.38)	
CC	6 (16.22)	8 (38.1)	
Age	47.49 ± 15.21	57 ± 16.12	**0.029**
HT			
No	19 (51.35)	13 (61.9)	0.616
Yes	18 (48.65)	8 (38.1)	
DM			
No	31 (83.78)	20 (95.24)	0.403
Yes	6 (16.22)	1 (4.76)	
CAD			
No	34 (91.89)	20 (95.24)	1.000
Yes	3 (8.11)	1 (4.76)	
Crea.	0.93 ± 0.17	0.96 ± 0.37	0.703
LDL	124.65 ± 28.2	108.52 ± 31.6	**0.049**
HDL	42.14 ± 9.6	41.43 ± 12.44	0.810
CRP	2.85 ± 1.65	4.16 ± 2.81	0.061
UA	5.25 ± 1.1	5.77 ± 1.51	0.134

HT: hypertension, DM: diabetes mellitus, CAD: coronary artery disease, Crea.: creatinine, HDL: high-density lipoprotein, LDL: low-density lipoprotein, CRP: C-reactive protein, and UA: uric acid.

**Table 2:** Echocardiographic findings of case and control groups.

	Control (*n* = 37)	Case (*n* = 21)	*p*
LAD	3.49 ± 0.18	4.75 ± 0.47	**<0.001**
AOD	3.25 ± 0.19	3.34 ± 0.14	0.057
LVSD	3.3 ± 0.27	4.28 ± 0.33	**<0.001**
LVDD	4.88 ± 0.24	5.41 ± 0.31	**<0.001**
LVEF	0.63 ± 0.04	0.55 ± 0.06	**<0.001**
IVST	1.14 ± 0.08	1.17 ± 0.1	0.224
PWT	1.14 ± 0.08	1.17 ± 0.1	0.156
MPAP	18.76 ± 2.09	40.29 ± 7.3	**<0.001**
MR grading			
Moderate		9 (42.86)	—
Severe		12 (57.14)	
Vena contracta width		0.65 ± 0.11	—

LAD: left atrial diameter, AOD: aortic root diameter, LVSD: left ventricular systolic diameter, LVDD: left ventricular diastolic diameter, LVEF: left ventricular ejection fraction, IVST: interventricular septal thickness, PWT: posterior wall thickness, MPAP: mean pulmonary artery pressure, MR: mitral regurgitation, and VCW: vena contracta width.

**Table 3:** Multiple logistic regression models for genotypes and other covariates associated with XPC.

	*β*	SE of *β*	Wald	*p*	OR	95% CI for OR	
						Lower	Upper
Genotype			6.77	0.034			
Genotype (AC)	3.27	1.30	6.34	**0.012**	26.37	2.07	336.73
Genotype (CC)	3.23	1.35	5.70	**0.017**	25.36	1.79	360.37
Age	0.13	0.05	8.58	**0.003**	1.14	1.04	1.24
HT	−2.12	1.08	3.81	0.051	0.12	0.01	1.01
DM	−3.17	1.62	3.80	0.051	0.04	0.00	1.02
CAD	−3.88	1.76	4.88	**0.027**	0.02	0.00	0.65
LDL	−0.05	0.02	6.83	**0.009**	0.95	0.91	0.99
Constant	−2.27	2.13	1.13	0.287	0.10		
